# Feeding Behavior of a Crab According to Cheliped Number

**DOI:** 10.1371/journal.pone.0145121

**Published:** 2015-12-18

**Authors:** Diogo Nunes de Oliveira, Ronaldo Adriano Christofoletti, Rodrigo Egydio Barreto

**Affiliations:** 1 Universidade Estadual Paulista “Julio de Mesquita Filho”, Instituto de Biociências (IBB-UNESP), Rubião Jr. s/n, 18618–970, Botucatu, São Paulo, Brazil; 2 Universidade Federal de São Paulo, Instituto do Mar, Campus Baixada Santista (IMar/UNIFESP), Av. Alm. Saldanha da Gama, 89 - Ponta da Praia; Santos (SP)–Brazil; 3 Departamento de Fisiologia, Instituto de Biociências, Caunesp, UNESP, Rubião Jr. s/n, 18618–970, Botucatu, São Paulo, Brazil; The Evergreen State College, UNITED STATES

## Abstract

Cheliped loss through autotomy is a common reflexive response in decapod crustaceans. Cheliped loss has direct and indirect effects on feeding behavior which can affect population dynamics and the role of species in the community. In this study, we assessed the impact of autotomy (0, 1, or 2 cheliped loss) on feeding behavior in the crab *Pachygrapsus transversus*, an omnivorous and abundant species that inhabits subtropical intertidal rocky shores along the South Atlantic Ocean. Autotomy altered crab feeding patterns and foraging behavior; however, the time spent foraging on animal prey or algae was not affected. These results indicate a plasticity of feeding behavior in *P*. *transversus*, allowing them to maintain feeding when injured.

## Introduction

Autotomy is an efficient reflexive response which results in loss of a limb at a pre-formed breakage plane [1–4]. This mechanism is considered a useful adaptation to avoid predators and limit injuries [5–9], occurring commonly in vertebrate and invertebrate groups; therefore, it has been investigated by several researchers [8, 10–12].

Despite the immediate survivorship benefits provided by autotomy [9, 13], the loss of one or more appendages may result in long-term energetic and functional costs [6, 8, 10, 11, 14, 15]. For example, crustaceans use chelipeds for defense, capture, manipulation, and subjugation of prey [16]. Damage or loss of those appendages can cause profound effects on feeding efficiency [17, 18], growth [19, 20], reproductive success [21, 22], alter the duration of each intermoult phase [18, 23], promote defective development [11], and creates limitations on the competitiveness of animals even after limb regeneration [24].

Especially in complex environments such as rocky shores where the diversity of predators and prey is high, nonlethal damage may influence population dynamics as well as community processes. Relatively few studies have examined the effects of autotomy on decapod crustaceans and knock-on consequences to the wider community, despite these animals being an important consumer in aquatic environments [8, 25–27].

On intertidal rocky shores of the Southwest Atlantic, the grapsid crab *Pachygrapsus transversus* (Gibbes, 1850) occurs in high abundance and can control prey populations and diversity. Its feeding habit is omnivorous, although when both algae and animal prey are available, there is preference for animal prey, but not for a specific animal group [28].

Following personal observations in the natural environment where individuals of *P*. *transversus* have been seen foraging even without both chelipeds, this study aims to describe and evaluate the consequences of cheliped loss onfeeding behavior. We assessed if crabs are able to feed themselves without chelipeds and if the foraging time and prey chosen change with the number of the chelipeds. Thus, results can contribute to our understanding of how autotomized crabs feed and survive and thus potentially the wider consequences of autotomy on prey populations.

## Materials and Methods

### Ethics statement

This research was authorized by the System of authorization and information on biodiversity (Sistema de Autorização e Informação em Biodiversidade—SISBIO, Brazil) from the Ministry of Environment of Brazilian government (Ministério do Meio Ambiente—MMA, Brazil), protocol number 30870–4.

### Crabs

*P*. *transversus* adults, with carapace width (CW) ranging from 13 to 25 mm, were collected during the spring season on rocky shores of the north coast of São Paulo State, Brazil. Crabs were classified in accordance to the number of chelipeds (0, 1, or 2) and, in the absence of sufficient specimens without cheliped, autotomy was induced in the laboratory by cutting the cheliped according to the methods previously described [29]. The gender and level of sexual maturity were not considered and analyzed in the present study based on previous observations showing no influence of these variables on stomach contents in this species (D. N. Oliveira unpubl. data). In this case, autotomized crabs were chosen randomly and the cheliped autotomized was also randomly chosen. After autotomization, crabs were maintained in individual tanks for 48 hours before starting the experiment.

### Experimental design and procedures

The experiment was carried out in two consecutive treatment blocks of 18 tanks and 12 randomized replications were performed in each treatment (0, 1 or 2 cheliped). For experimentation, crabs were acclimated individually into aquaria with a central shelter and flowing sea water for 48 hours fasting. Food types were chosen based on previous analysis of stomach contents of this species [28], which determined the most consumed prey. After this acclimatization period, 7 cm^2^ each food type (ascidians, the bivalve *Brachidontes solisianus* (d'Orbigny, 1842), biofilm, and macroalgae) were arranged in parallel in a randomly chosen corner in each aquarium. The bivalve *B*. *solisianus* had a length ranging from 0.34 to 1.72 cm. Only intact organisms were used as prey to avoid any influence of chemical cues. All food items were washed in sea water and visually examined to remove any other organisms that could serve as a food source.

**Fig 1:**
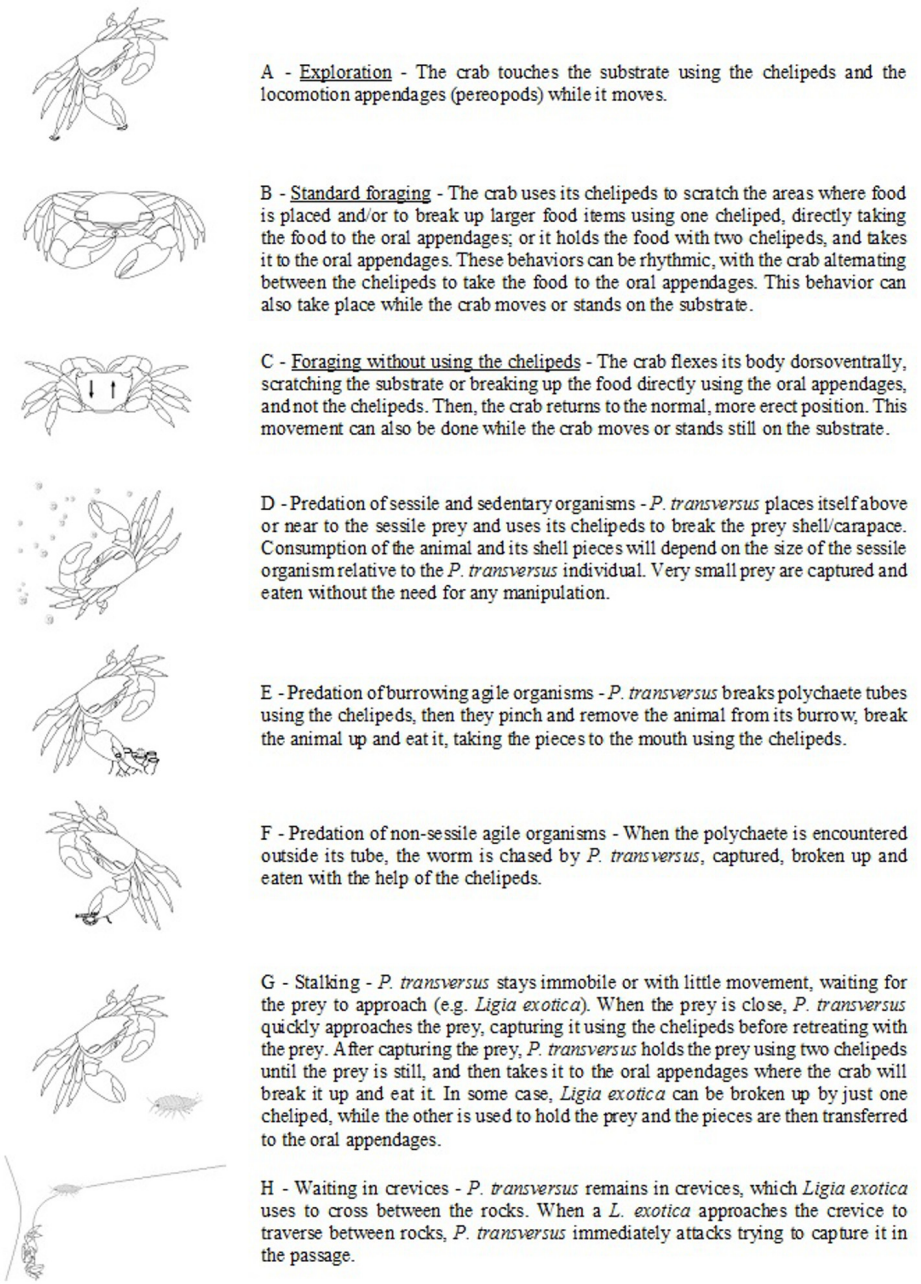
Ethogram of the feeding behavior of *P*. *transversus* observed in the natural environment and in the laboratory.

**Fig 2:**
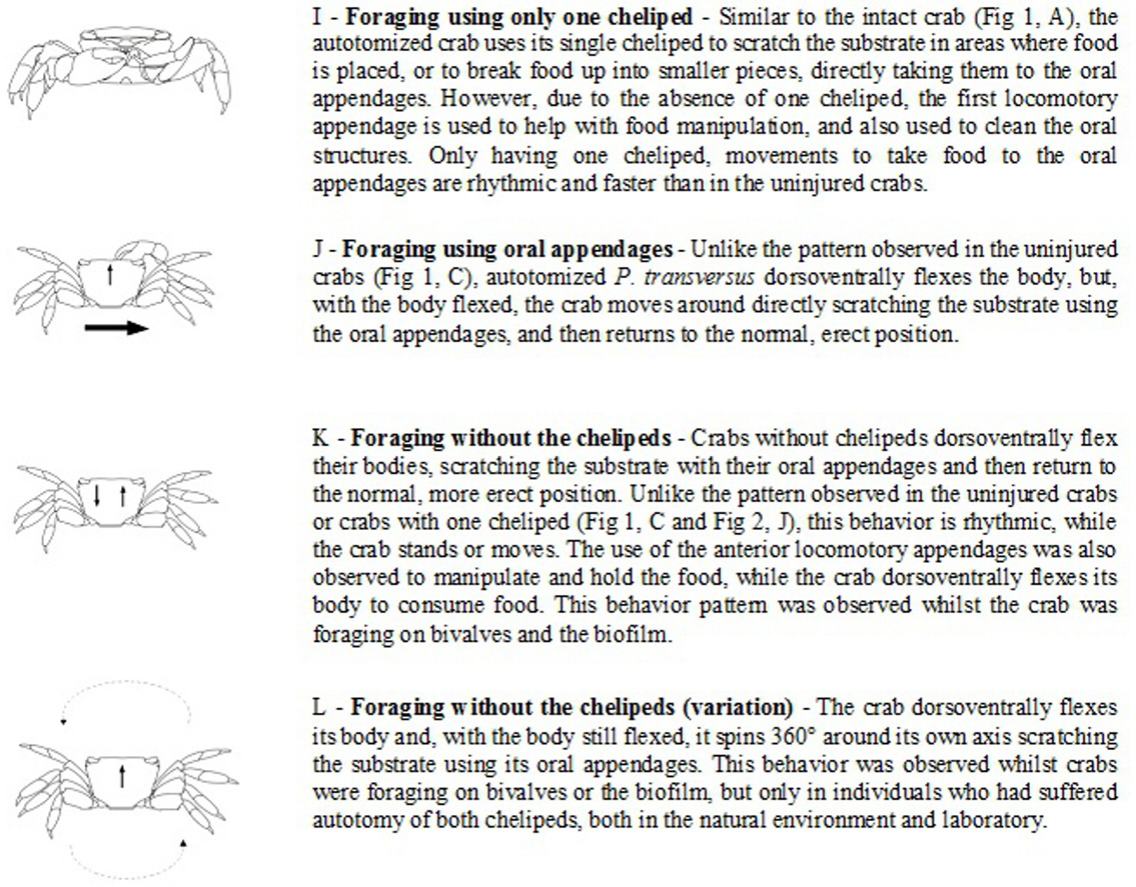
Ethogram of the feeding behavior of autotomized *P*. *transversus* observed in the natural environment and in the laboratory.

Crabs were monitored using digital cameras for one hour to quantify the time spent foraging. Foraging time was determined as the ratio of time spent handling a specific food to the total time of filming, a procedure based on [28]. Foraging time was considered as the time that crabs spent holding a food item and taking the chelipeds to the oral appendages (Figs 1B and 2I). For crabs without chelipeds, foraging time consisted of the period during which the crab performed the movement to flex the body dorsoventrally by putting oral appendages in contact with food on substract (Fig 2K–2L).

Ethograms of feeding behavior as a function of the number of chelipeds were described for *P*. *transversus* foraging on sessile prey based on two laboratory filming periods of 36 and 99 hours. Furthermore, 21 hours of film, pictures, and in situ observations of predation behavior of *P*. *transversus* on mobile prey such as polychaetes and the isopod *Ligia exotica* (Roux, 1828) were added.

### Statistical analysis

Data on foraging activity rate (dependent variable) were arcsine transformed. Following transformation, data were normally distributed and homoscedastic according to the Kolmogorov-Smirnov test and Levene’s test, respectively. Data were analyzed using a two-way mixed ANOVA (one-between-subjects categorical predictor and one-within-subjects repeated measures ANOVA) followed by a Newman–Keuls post-hoc test, with number of claws as independent factor and food types as repeated measures. Statistical differences were considered significant when P < 0.05.

## Results

### Feeding behavior

The first activity of *P*. *transversus* during foraging is exploration of the environment touching the substrate with chelipeds and/or pereopods to find the food source. Observations show that autotomy does not have an influence on this behaviour (Fig 1A and 1D). Crabs with 1 or 2 chelipeds used them as their principal feeding tool, using the cheliped to tear the food and then to eat it (Figs 1B and 2I).

Furthermore, crabs without chelipeds used the oral appendages as the principal feeding tool. Crabs without chelipeds flex their body dorsoventrally, lowering the body down to the substrate, scratching and breaking up the food by using the oral appendages (Fig 2K–2L). This behavior was observed for both uninjured and autotomized crabs, but for uninjured crabs, it was less frequent and lasted only for a short period of time before the body was returned to the erect position (Figs 1C and 2J). Predatory behavior on mobile organisms (e.g., the isopod *L*. *exotica* and polychaetes) were only observed in uninjured *P*. *transversus* specimens *in situ* (Fig 1E–1H).

### Foraging activity

**Fig 3:**
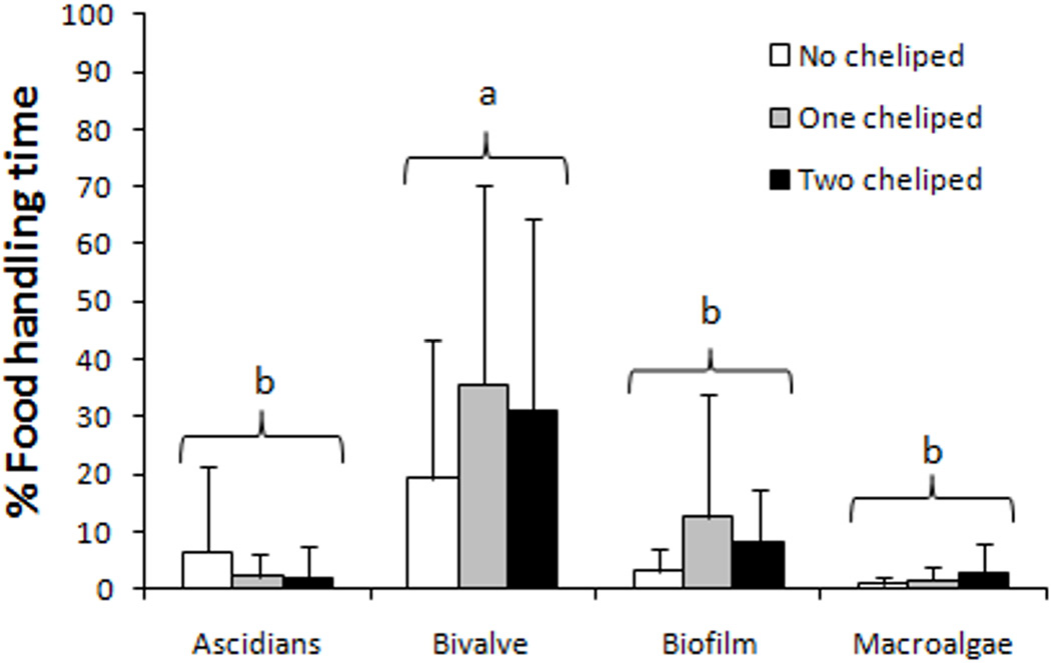
Foraging time (%, mean ± SD, see S1 Table for details) in relation to food type and cheliped number in *P*. *transversus*. Different letters represent significant differences among food types (two-way repeated measures ANOVA; p < 0.05).

Autotomy neither influenced the foraging activity (F_(2;33)_ = 1.26; P = 0.297), nor the interaction with food types (F_(6;99)_ = 0.98; P = 0.443). However, crabs foraged significantly more on the bivalve *B*. *solisianus* compared to other food types (F_(3;99)_ = 17,36; P < 0.00001; Fig 3) regardless of the number of chelipeds.

## Discussion

Autotomy had no influence on foraging time, because the crab *P*. *transversus* showed clear behavioral plasticity in their feeding pattern according to the number of chelipeds, which allow crabs to handle different food items. Change in feeding behavior is an adaptive mechanism which enables crabs to continue feeding on the same type of prey.

The number of chelipeds present influenced the activity patterns of *P*. *transversus* and reduced their behavioral repertoire. The lower the number of chelipeds, the smaller the behavioural repertoire became. All autotomized crabs used locomotor appendages to capture and manipulate food during foraging. This behavior has also been observed for *Menippe mercenaria* [30], *Callinectes sapidus* [17, 31], *Hemigrapsus sanguineus* [32], and for the congener *Pachygrapsus crassipes* [33, 34]. Thus, we suggest that the use of locomotor appendages is a compensatory mechanism to overcome the absence of chelipeds in crustaceans, allowing them to maintain nutrient acquisition for essential biological processes, such as molt and regeneration of a new limb. However, this issue was not the aim of the present study and must be assessed in future studies.

Feeding behavior of *P*. *transversus* showed that autotomy influences the way the crabs feed but does not influence food choice. Bivalves were previously identified as the preferred prey of this crab [28] corroborating with the present study where crabs spent significantly more time handling bivalve prey instead of algae, biofilm, or ascidians, regardless of the number of chelipeds. A predator does not need a second cheliped to hold a prey, to open or crush the shell, explaining why there is no change in food preference with loss of chelipeds [35].

In other crab species, autotomy also showed no effect on time spent foraging but feeding rate decreased in *C*. *pagurus* [29], *Cancer magister* [34], *Hemigrapsus sanguineus* [32] and *Carcinus maenas* [35]. In the present study, we did not quantify feeding rate, but we observed shell fragments and bivalves without some parts in all treatments, indicating that mussels had been ingested independently of the number of chelipeds.

Some behaviors are considered patterns and are well known for decapods. For example, the behavior of manipulating food with the locomotor appendages was observed here in all three treatments (0, 1, and 2 chelipeds), although mainly in autotomized crabs. Such behavior has also been reported for other species [17, 30–33]. In this study we specifically observed the behavior in which the crabs flexed dorsoventrally their body and rotated 360° around their own axis, in order to manipulate the food with the oral appendices. This behavior was observed for crabs that had autotomized both chelipeds and allowed feeding to occur.

Our study demonstrated that autotomy has the capability of modulating behavioral changes in *P*. *transversus*, but autotomy is not a limitation large enough to modify the food preferences of this species. Also, a new behavior was observed in crabs with both chelipeds autotomized that allowed crabs to retain feeding capacity. Further studies on the use of locomotor appendages as a compensatory mechanism to overcome the absence of chelipeds in crustaceans are warranted.

## Supporting Information

S1 Table Foraging time (%, mean ± SD) in relation to food type and cheliped number in *P*. *transversus*. (DOC)
