# Supplementary material for: Feeding Behavior of a Crab According to Cheliped Number
Source: PLoS One. 2015 Dec 18;10(12):e0145121. doi: 10.1371/journal.pone.0145121 (PMC4690604; doi:10.1371/journal.pone.0145121)
Supplement: S1 Table — (DOC) [file pone.0145121.s001.doc]

**Feeding behavior of a crab according to cheliped number**

S1 Table. Foraging time (%, mean ± SD) in relation to food type and cheliped number in *P. transversus*.

|  | Ascidian | |  | Brachidontes | |  | Biofilm | |  | Macroalgae | |
| --- | --- | --- | --- | --- | --- | --- | --- | --- | --- | --- | --- |
|  | Mean | SD |  | Mean | SD |  | Mean | SD |  | Mean | SD |
| 0 cheliped | 0,064 | 0,148 |  | 0,193 | 0,241 |  | 0,032 | 0,037 |  | 0,008 | 0,011 |
| 1 cheliped | 0,023 | 0,038 |  | 0,356 | 0,348 |  | 0,125 | 0,215 |  | 0,015 | 0,024 |
| 2 cheliped | 0,019 | 0,057 |  | 0,310 | 0,335 |  | 0,083 | 0,092 |  | 0,026 | 0,051 |
